# Erratum for Soto-Aceves et al., “The relationship between *pqs* gene expression and acylhomoserine lactone signaling in *Pseudomonas aeruginosa*”

**DOI:** 10.1128/jb.00475-24

**Published:** 2024-12-11

**Authors:** Martín P. Soto-Aceves, Nicole E. Smalley, Amy L. Schaefer, E. Peter Greenberg

## ERRATUM

Volume 206, no. 10, e0138-24, 2024, https://doi.org/10.1128/jb.00138-24. Page 6, last line: "*pqsA1*" should read "*phzA1.*"

Page 7, Table 2: the gene name *phzA^C^* should be associated with locus PA1899.

Page 10, line 19: "*pqsA1*" should read "*phzA1.*"

